# Microsatellite Loci Reveal Genetic Diversity of Asian Callery Pear (*Pyrus calleryana*) in the Species Native Range and in the North American Cultivars

**DOI:** 10.3390/life11060531

**Published:** 2021-06-07

**Authors:** Shiwani Sapkota, Sarah L. Boggess, Robert N. Trigiano, William E. Klingeman, Denita Hadziabdic, David R. Coyle, Bode A. Olukolu, Ryan D. Kuster, Marcin Nowicki

**Affiliations:** 1 Department of Entomology and Plant Pathology, University of Tennessee, Knoxville, TN 37996, USA; ssapkot1@vols.utk.edu (S.S.); sbogges1@vols.utk.edu (S.L.B.); rtrigian@utk.edu (R.N.T.); dhadziab@utk.edu (D.H.); bolukolu@utk.edu (B.A.O.); rkuster@utk.edu (R.D.K.); 2 Department of Plant Sciences, University of Tennessee, Knoxville, TN 37996, USA; wklingem@utk.edu; 3 Department of Forestry and Environmental Conservation, Clemson University, Clemson, SC 29634, USA; dcoyle@clemson.edu

**Keywords:** ‘Bradford’ pear, SSR, introduced horticultural tree, woody ornamental, invasive species

## Abstract

*Pyrus calleryana* Decne. (Callery pear) includes cultivars that in the United States are popular ornamentals in commercial and residential landscapes. Last few decades, this species has increasingly naturalized across portions of the eastern and southern US. However, the mechanisms behind this plant’s spread are not well understood. The genetic relationship of present-day *P. calleryana* trees with their Asian *P. calleryana* forebears (native trees from China, Japan, and Korea) and the original specimens of US cultivars are unknown. We developed and used 18 microsatellite markers to analyze 147 *Pyrus* source samples and to articulate the status of genetic diversity within Asian *P. calleryana* and US cultivars. We hypothesized that Asian *P. calleryana* specimens and US cultivars would be genetically diverse and would show genetic relatedness. Our data revealed high genetic diversity, high gene flow, and presence of population structure in *P. calleryana*, potentially relating to the highly invasive capability of this species. Strong evidence for genetic relatedness between Asian *P. calleryana* specimens and US cultivars was also demonstrated. Our data suggest the source for *P. calleryana* that have become naturalized in US was China. These results will help understand the genetic complexity of invasive *P. calleryana* when developing management for escaped populations: In follow-up studies, we use the gSSRs developed here to analyze *P. calleryana* escape populations from across US.

## 1. Introduction

Genetic diversity and population structure of any species are important factors for determining the long-term survival of the species and their adaptation to environmental changes [1]. The population genetic profile of a species can inform about the origins of sub-populations [2,3], which provides greater understanding about the introduction history of the tree and its cultivars. Such knowledge can be used to formulate an effective management plan. Assessment of the existing genetic variation of any species requires widespread and intensive sampling from both native and introduced ranges [4].

*Pyrus calleryana* Decne., Callery pear, is a species of pear tree native to eastern and southern China, Taiwan, Korea, and Japan [5]. This deciduous tree often has a conical to rounded crown and flowers as early as three years of age [5,6]. *Pyrus calleryana* is a popular ornamental tree well-known for its early spring flowers, robust growth, and fall color display [7]. ‘Bradford’ is the most widely planted and the most commonly known Callery pear cultivar in the United States (USA) [8,9]. *Pyrus calleryana* is diploid with a haploid chromosome number of 17, 2*n* = 34 [6] and flow cytometry estimated genome size of 588 Mbp/1C [10]. Flowers from the cultivars of *P. calleryana* are self-incompatible and cultivars are routinely vegetatively propagated by grafting. The trees can produce viable, long-lived seeds [11], when flowers are cross-pollinated by other compatible *Pyrus* species, compatible rootstock-based pollen, or other *P. calleryana* cultivar [12]. When this occurs, the seeds can germinate and establish when dispersed in favorable environments [9]. In addition, *P. calleryana* adapts well to soils of variable pH (acidic to alkaline soil) and trees are tolerant of drought [7]. Several traits, including gametophytic self-incompatibility, pathogen and herbivore resistance, and tolerance of various abiotic stresses have contributed to the spread and persistence of *P. calleryana* in a variety of environments [12]. Although the underlying mechanisms and processes that have enabled the broad spread and invasiveness of the species are not well understood, intraspecific hybridization among the genetically distinct cultivars and interspecific hybridization with the other escaped *Pyrus* species could be among the possible reasons behind expansion of *P. calleryana* populations [7,13]. Additionally, several insects promote cross-pollination [8,14]. The fruits on fertile naturalized trees become secondary foods for birds that then disperse seeds into distant areas, and contribute to the spread of the species [15].

Major imports of *P. calleryana* seeds to the USA were made from East Asia in 1917 through 1920 and that germplasm was used to breed with European pear, *P. communis* L. Selections were performed based on resistance against the fire blight pathogen, *Erwinia amylovora* Burrill [12]. The hardiness of *P. calleryana* lent itself to the development and release of several intraspecific hybrid cultivars making it popular among local gardeners and landscapers. Soon, *P. calleryana* appeared in and naturalized almost all habitats throughout the USA [8,16,17,18,19]. Naturalized *P. calleryana* trees can be found in 33 states [16,17] and due to expanding populations of naturalized trees, *P. calleryana* has been listed, or watch-listed, as an invasive species in many USA states [9,12,20]. *Pyrus calleryana* can potentially become one of the most problematic invasive species in the USA [21]. Hence, there is need for understanding genetic contributions to invasiveness as a means to develop effective management plans and strategies that can mitigate or limit the spread of *P. calleryana*. To address this knowledge gap, we need to know more about the biology and genetics of this invasive species.

The assessment of the molecular population genetics preferably using co-dominant markers is necessary for understanding the dynamics of adaptation and the spread of any species [22]. The use of simple sequence repeats (microsatellites; SSRs) in population genetics and phylogenetics provides a more reliable interpretation of genetic diversity than other arbitrary markers such as random amplified polymorphic DNAs (RAPDs) [23,24]. SSRs, highly informative and multi-allele genetic markers, are experimentally reproducible and transferable among closely related species [25]. The presence of multiple alleles at an SSR locus makes SSRs more informative than other molecular markers, including SNPs [26].

Most of our knowledge on *P*. *calleryana* originates within its native range in Asia, where *P*. *calleryana* naturally occurs as scattered individuals in the wild to the point of being considered a threatened species [14,27]. Most of these studies have focused on identification and characterization of cultivars or *Pyrus* species using different types of DNA markers such as SSRs [28,29,30,31], amplified fragment length polymorphisms (AFLPs) [32], RAPDs [33], restriction fragment length polymorphisms (RFLPs) [34], and chloroplast DNA (cpDNA) [35]. Few studies, however, have investigated the genetic diversity and population structure of *P*. *calleryana*. Nuclear SSRs (nSSRs) and cpDNA were used in a study of genetic diversity of *P*. *calleryana* in Zhejiang province of China [36], which revealed the geographic distance as the major factor in shaping the population structure of *P*. *calleryana*. Additionally, an examination of the genetic diversity of *P*. *calleryana* var. *dimorphophylla* in the Tokai district of central Japan [7,27,37] found complex genetic structure of the species, probably originating from artificial propagation and introgression with other *Pyrus* species. There are limited studies investigating the genetic diversity and population structure of the invasive USA *P. calleryana* escapes, and this issue urgently needs further investigation [7,37].

We assessed the genetic diversity and population structure of *P*. *calleryana* from both native (i.e., Asian) and introduced ranges (the USA-introduced cultivars). We hypothesized that there would be high genetic diversity present within Asian *P. calleryana* populations, and that they would be genetically related to early USA-naturalized cultivars of *P. calleryana* (owing to the origin of the species) in terms of genetic distance and their genetic clustering. Specifically, we aimed to (a) develop novel microsatellite markers for *P*. *calleryana*, (b) test the cross-species amplification of the developed genomic short sequence repeats (gSSRs) to other *Pyrus* and *Malus* species (a related outgroup, of economic importance), and (c) use the most informative of these markers to evaluate the genetic diversity and genetic relatedness among Asian *P. calleryana* specimens and early USA-naturalized cultivars of *P*. *calleryana*.

## 2. Materials and Methods

### 2.1. Sample Collection

Leaf and flower bud samples of pears (*Pyrus*) and the original USA cultivar selections of *P*. *calleryana* were obtained from 10 herbaria and arboreta in the USA. In total, 147 samples (including 80 samples of *P*. *calleryana* and 67 samples of other *Pyr* and *Malus* species) were received. Respective permissions or licenses for limited destructive sampling were granted by all these institutions, in accordance with their internal regulations. The geographical coordinates, the country of origin, and the year of collection were recorded for each sample and whenever possible for historical specimens [38]. The collection was reduced to 90 specimens due to the unreliable/inconsistent amplification of samples. Out of those 90 samples, 57 were *P. calleryana* specimens (36 specimens of Asian *P*. *calleryana* and 21 samples of 7 USA commercial cultivars of *P*. *calleryana*) and 33 samples represented 14 different species of *Pyrus* and 2 samples of *Malus rockii* (Supplementary Table S1). These 33 *Pyrus* and *Malus* species samples were used to evaluate the potential for cross-amplification among gSSRs.

### 2.2. gDNA Extraction

Approximately 100 mg of dried leaf samples or fresh flower buds were taken from each specimen and homogenized using a Bead mill 24 (Fisher Scientific, Pittsburgh, PA, USA). Samples were homogenized four times and kept frozen in liquid nitrogen for at least 5 min between each homogenization step in order to improve the tissue homogenization. The gDNA was extracted using EZNA DNA DS Mini Kit (Omega Bio-Tek, Norcross, GA, USA) protocol. Nanodrop (Thermo Fisher Scientific, Wilmington, DE, USA) was used to evaluate the purity and measure the concentration of the isolated gDNA. Re-extraction was attempted following the CTAB protocol [39] for those samples that did not produce good quality and quantity of gDNA using EZNA DNA DS Mini Kit. gDNAs was re-extracted wherever enough of plant sample material was available.

### 2.3. Microsatellite Primers and Genotyping Conditions

Genomic SSRs (gSSRs) were developed by using closely related pear (*Pyrus × bretschneideri* Rehder) whole genome sequence data (GenBank number: JH994112) [40]. The development of gSSRs involved several steps including: (i) genome assembly using MaSuRCA [41]; and (ii) SSR mining using GMATA [42]. The gSSRs of interest with a motif length of 2 to 6 bp and a minimum of 5 repeats were retained. Finally, 18 to 22 bp gSSR primers were designed using Primer3 [43] with 45 to 55% GC content, 58 to 62 °C melting temperature, and 100 to 500 bp of expected product sizes. The developed gSSRs were used for this *P*. *calleryana* study (IDT, Coralville, IA, USA; Supplementary Table S2). Genomic locations of the gSSRs used in this study were analyzed in six available genomes of related *Prunus* spp. using BLAST [44] with default settings, owing to their relatively higher quality (contiguity, N score, and annotation) than the genome used for their development. Genome with most gSSRs mapping to it was used to visualize their locations. Additionally, for cross-species amplification analysis, gDNA samples of the other *Pyrus* species that failed to amplify with the gSSRs were further tested with Internal transcribed spacer (ITS) primers [45] and ribosomal protein S16 (*rps*16/cpDNA03) primers [46]. This was done to confirm that the failure of gSSR-PCR was not due to inferior quality of gDNA used. Both ITS (nuclear) and *rps*16 (plastidic) primers were used in our study for more effective DNA quality controls.

Polymerase chain reaction (PCR) amplifications were performed in a 10 μL reaction mixture consisting of 4 ng gDNA, 1 μM final concentration of each primer, 5 μL of 2 × GoTaq^®^ DNA Polymerase (Promega, Madison, WI, USA), and 0.5 μL of dimethyl sulfoxide (DMSO). In order to validate the data, the gDNA extracted from the *P. calleryana* herbarium specimen (Arnold Arboretum, catalog number: 156,119) was used as a positive control, and sterile distilled water as a negative control, for each of 18 gSSRs tested. PCR amplification was performed using the touch-down protocol to ensure the specificity of the amplified fragments [47] using the following thermal profile: initial denaturation at 94 °C for 3 min., followed by 10 cycles of denaturation at 94 °C for 30 s, annealing at 65 °C for 30 s with a touch-down of 0.7 °C/cycle and an extension at 72 °C for 30 s then, followed by 30 cycles of denaturation at 94 °C for 30 s, annealing at 58 °C for 30 s and an extension at 72 °C for 30 s, with a final extension of 72 °C for 4 min.

QIAxcel Capillary Electrophoresis System (QIAGEN, Germantown, Maryland, USA) was used to visualize the *P*. *calleryana* PCR products and determine allelic sizes using a 15/600 bp alignment marker and 25 to 500 bp DNA size marker. Allele sizing was performed for each of the 147 gDNA samples against each of the 18 gSSR markers. The PCR reaction was repeated twice for the samples that did not amplify, which were then declared missing data if still failed to amplify. Samples with missing data in more than 9 of 18 loci were discarded from the analyses resulting in the final dataset of 57 *P*. *calleryana* samples (Supplementary Table S3 and data not shown).

For cross-species amplification test, PCR amplification in the gDNA samples of the other *Pyrus* and *Malus* species was also performed using this PCR reaction mixture composition and following the touch-down protocols detailed above. In addition, PCR amplification for the samples that failed to amplify for each gSSR was further attempted using ITS [45] and *rps*16 primers [46] using a 10 μL reaction mixture: 10 ng gDNA, 5 μM final concentration of each primer (ITS or *rps*16, respectively), and 5 μL of AccuStart II PCR SuperMix (Quantabio, Beverly, MA, USA). *Pyrus calleryana* (Carnegie Herbarium, catalog number: 396078) DNA was used as a positive control and sterile distilled water was used as a negative control.

The optimized thermal profile for PCR with ITS1 and ITS4 primers included an initial denaturation at 94 °C for 2 min, 40 cycles of 95 °C for 30 s, 60 °C for 1 min, 72 °C for 90 s, and the final extension at 72 °C for 7 min. The thermal cycle for touchdown PCR involving *rps*16 primers included the following settings: 95 °C for 4 min, 95 °C for 20 s, 58 °C for 30 s (−0.3 °C/cycle), (72 °C for 90 s) for 10 cycles; (95 °C for 20 s, 55 °C for 30 s, 72 °C for 90 s) for 35 times, and 72 °C for 5 min. The products of the PCR reactions were electrophoresed using 2% *w*/*v* agarose gels with ethidium bromide stain at 100 V/cm^2^ for 1 h, visualized under UV light using UVP GelStudio PLUS (Analytikjena, Upland, CA, USA) and documented using VisionWorks 8.22.18309.10577 (Analytikjena, Upland, CA, USA).

### 2.4. Data Analysis

The raw allele sizes were binned into statistically identical allelic classes using FlexiBin excel macro [48]. The binned allelic data were used for further analyses. For each gSSRs, the *P*. *calleryana* dataset was transformed using the motif lengths to represent the number of repeats rather than the PCR allele sizes using PGDSpider [49] version 2.1.1.5 (University of Bern, Bern, Switzerland). Clone correction was completed using *poppr* [24] version 2.8.5 (Oregon State University, Corvallis, OR, USA) in RStudio version 1.2.5033 (RStudio, Boston, MA, USA) using R [50] 3.6.2 (R Foundation, Vienna, Austria). There were no clones in the dataset (total *n* = 57). Based on their country of origin, the samples were divided into four population groups, denoted “China” (*n* = 20), “Japan” (*n* = 11), “Korea” (*n* = 5), and “USA” (*n* = 21).

### 2.5. Population Genetics of P. calleryana

#### 2.5.1. Genetic Diversity

The genetic diversity indices across the 18 gSSRs and 4 population groups were calculated using R with packages *poppr* version 2.8.5 and *hierfstat* [51] version 0.04-22, and SPAGeDi [52] version 1.5 (Free University of Brussels, Brussels, Belgium). For each gSSR marker, the number of alleles amplified (N), observed heterozygosity (H_o_), expected heterozygosity (H_e_), Jost’s differentiation estimate (D_est_), Stoddard and Taylor index (G), and allelic richness (A_r_) were calculated using *hierfstat*. SPAGeDi was used to estimate the presence of private alleles (P_a_), as well as hierarchical fixation indices including inbreeding coefficients (F_IS_, R_IS_), allele fixation index (F_ST_), and R_ST_ (F_ST_ analogue based on allele size) [53,54]. Gene flow (N_m_ = ¼ × [(1/F_ST_) − 1]) among populations was estimated using GenAlEx [55] version 6.5 (Rutgers University, New Brunswick, NJ, USA).

In order to assess the contribution of mutation rate to the population structure, SPAGeDi performed the permutation tests to determine the phylogenetic distance between individuals using 10,000 permutations among alleles within each locus and to determine the significance of inbreeding coefficients using 10,000 permutations among gene copies [56]. For many samples, including many of the historical specimens, Global Positioning System (GPS) coordinates were not available, shrinking the sample subset further, and consequently creating a possible bias in our study. The entire *P*. *calleryana* dataset (*n* = 57) was used to determine the phylogeographical signal within populations as R_ST_ does not depend on GPS coordinates, whereas, only the individuals with known GPS coordinates of origin (total *n* = 28; “China”, “Japan”, “Korea” groups) were used to determine the phylogeographical signal among populations.

Analysis of Molecular Variance (AMOVA) was performed using the package *poppr* with 1000 permutations in order to evaluate the molecular variance partitioning within and among the population groups. Linkage Disequilibrium (LD) of the used gSSRs was determined in *poppr*, with significance assessed using 1000 permutations. The pairwise index of association ($\bar{r}$_d_) was calculated using permutation approach to assess whether the loci were linked and to ensure that the observed pattern of LD is not due to a single pair of loci [57].

#### 2.5.2. Population Structure

##### Mantel Test for Isolation by Distance

Samples with known GPS coordinates (*n* = 28; Supplementary Figure S1) were analyzed using Mantel and partial Mantel tests implemented in the package MASS [58] version 7.3-50 (CSIRO, Cleveland, Australia) using 1000 permutations for the assessment of tests significance. These tests estimate isolation by distance (IBD) to determine the correlation between the genetic and the geographical distance matrices of the individuals. The Mantel test was also standardized using the year of sampling (partial Mantel test), to additionally assess the effect of mutation across time. Further, Mantel correlogram tests were performed to further examine the underlying correlative relationship using the packages *ade4* [59] version 1.7-13 (Université Claude Bernard Lyon 1, Lyon, France) and *vegan* [60] version 2.5-3 (University of Helsinki, Helsinki, Finland) using α = 0.05.

##### Bayesian Clustering Using Structure and DAPC

Structure  [61] version 2.3.4 (Stanford University, Stanford, CA, USA) was used to analyze the population structure of the *P. calleryana* dataset utilizing a Bayesian clustering algorithm. Genetic clusters among the *P. calleryana* individuals were inferred using 30 independent Monte Carlo Markov Chains (MCMC) with 250,000 generations of burn-in period and 750,000 MCMC steps in the actual runs for each used number of clusters (K = 1 to 10). Structure results were then visualized with PopHelper [62] version 1.0.10 (Uppsala University, Uppsala, Sweden) using the Evanno’s method [63]. ObStruct [64] version 1.0 (University of Auckland, Auckland, New Zealand) was used to determine the correlation of population structure of inferred ancestral profiles to that of predefined/sampled populations. The program uses the ad hoc R^2^ statistics whose values range from 0 (recent divergence of predefined populations or a lot of migration/admixture between populations) to 1 (strong diversification and/or population structure) [64]. One-tailed t-tests were used to accrue the significance of the differences when consecutively removing each one of the pre-defined populations or the inferred clusters, to assess this group’s impact on R^2^.

The population structure of the *P. calleryana* dataset was also analyzed using model-free multivariate clustering approach, Discriminant Analysis of Principal Components (DAPC) using the package *adegenet* [65] version 2.1.1 (Université de Lyon, Lyon, France). The DAPC analysis was optimized and cross-checked utilizing 1000 permutations over Principal Component Analysis (PCA) range from 2 to 109 (total number of alleles – 1). The analysis was further confirmed using a dendrogram of unrooted neighbor-joining tree of pairwise genetic distances [66] among *P*. *calleryana* originating populations. A separate DAPC analysis was also performed for USA cultivars only.

## 3. Results

### 3.1. gSSR Development and Selection

After gSSR mining with GMATA and execution of additional in-house scripts, a total of 115,838 SSRs were discovered from three *Pyrus × bretschneideri* draft genomes. Marker polymorphism was determined based on allelic variation within each genome or across the three genome assemblies. SSR mining and primer design resulted in 105,557 SSRs with acceptable primers and of these, 90,987 were dinucleotide, 10,913 were trinucleotide, and 2891 were tetranucleotide repeats. Primers between 59 to 60 °C with <1 °C difference between forward and reverse primers melting temperatures resulted in the discovery of 15,269 single motifs monomorphic SSRs along with 306 single motifs polymorphic SSRs. Only single motifs polymorphic SSRs were considered for the further analysis. In the single motifs polymorphic SSRs collection, AG was the most frequent dinucleotide motif and AAG was the most frequent trinucleotide motif (Supplementary Figure S2a). For the study, 50 gSSRs were selected and tested using gDNA samples from three locally escaped, naturalizing Callery pear trees (Third Creek Greenway, Knoxville, TN, USA). Based on preliminary data and the resulting amplification robustness, polymorphic character, and agreement with the expected product sizes, 40 gSSRs were selected for further evaluations. Of these, based on the initial assessment, 18 gSSRs with high discriminating power for multi-locus genotypes of *P*. *calleryana* gDNA were included in the subsequent analyses (Supplementary Table S2). Genomic locations of those 18 gSSRs were analyzed using the six available high-quality genomes of related *Prunus* spp. Among those, 17 gSSR mapped to *Prunus dulcis* (Mill.) D.A.Webb genome GCF_902201215.1 [67] (Supplementary Figure S2b).

### 3.2. Cross-Amplification

Cross-amplification was performed using 18 gSSRs (Supplementary Table S4). Within the genus *Pyrus*, all the gSSRs amplified in three *Pyrus* species: *P*. *communis*, *P*. *longipes*/*cossonii*, and *P*. *pyrifolia*. Likewise, the gSSRs cross-amplified at high rates in *P*. *pashia* (94%) and *P*. *amygdaliformis* (89%). The developed gSSRs performed well in *Malus rockii* (67%). Additionally, the gDNA samples that failed to amplify in *P*. *gharbiana*, *P*. *korshinskyi*, *P*. *regelii*, and *P*. × *hybrid* (‘Bartlett’ × *P*. *salicifolia*) samples using our gSSRs were successfully amplified using both ITS and *rps*16 primers (Supplementary Figure S3).

### 3.3. Population Genetics of P. calleryana

#### 3.3.1. Genetic Diversity

All 57 individual *P*. *calleryana* samples represented unique MLGs and their genotypic data were used for analyses. Throughout the dataset, there was about 10% missing data (Table 1). The locus and population with the highest missing data were PyC012 with 26%, and “China” with 15% missing data, respectively. The dataset deviated from Hardy–Weinberg equilibrium (HWE; Supplementary Figure S4), which is a possible result from the relatively low sample number, from specimens that were sampled across a broad time range (approximately 1912 to 2019 AD), as well as from wide geographical origins.

Among the four populations, the average Shannon-Wiener Index of MLG diversity (H) was 4.04, indicating high genetic diversity in the genotypic dataset (Table 1). The average effective number of alleles in the four populations was 5, ranging from 2 in “Korea” to 6 in “China”. This indicated high genetic diversity and variations of *P*. *calleryana* populations. Similarly, the overall A_r_ of 3.79 varied from 2.48 in “Korea” to 4.61 in “China”. A total of 88 private alleles were found in the *P*. *calleryana* dataset, with “China” population having the most of private alleles (*n* = 37).

The gSSRs had high power in discriminating the MLGs requiring only 8 gSSRs to capture all of the MLGs present (Supplementary Figure S5). The calculation of a small range of pairwise values of LD ($\bar{r}$_d_ = 0 to 0.4, *p*-value = 0.113) indicates no linked loci and suggests distribution of gSSRs across the genome (Supplementary Figure S6).

Across the dataset, an average of about 12 alleles per locus (ranging from 5 to 20) were detected (Table 2). The mean allelic richness (A_r_) calculated in the locus-wise manner was 4.70, ranging from 2.74 (PyC050) to 6.22 (PyC031), suggesting a high long-term adaptability and persistence potential of the *P*. *calleryana* populations. There was a moderate observed heterozygosity across 18 gSSRs overall (H_o_ = 0.34) ranging from 0.03 (PyC050) to 0.93 (PyC038). In addition, there was a high expected overall heterozygosity (H_e_ = 0.81) across all loci ranging from 0.41 (PyC050) to 0.92 (PyC031 and PyC017). The overall H_o_ < H_e_ implied the presence of population structure within our *P*. *calleryana* collection. Furthermore, the dataset indicated high gene flow across all gSSRs with an overall value of 1.79.

For the assessment of phylogeographic signals within the *P*. *calleryana* dataset, SPAGeDi was implemented, using 10,000 permutations among alleles within each locus. The mean permuted R_ST_ over all loci was not statistically different from F_ST_ in accordance with the expectations of this test, and the observed R_ST_ was bigger than the observed F_ST_ (*P*obs > exp = 0) indicating the presence of phylogeographic signal within populations (data not shown). Furthermore, the slope test of pairwise R_ST_ was evaluated in both linear and logarithmic forms to assess the phylogeographic signal among populations. From this slope test, we found no evidence of phylogeographic signal among populations (*P*obs > exp = 0.95). Only the samples with available GPS coordinates (*n* = 28) were used for the slope test, thus creating a possible bias in this analysis.

AMOVA was used to investigate the partitioning of the molecular variance. It suggested a low proportion of total molecular variance among populations (6.2%), with more than half of the total molecular variance partitioned within individuals (63.5%) (Supplementary Table S5). Significance of the test result (*p* < 0.001) indicated the existence of population structure within the genotyped collection of *P*. *calleryana*.

#### 3.3.2. Population Structure

##### Mantel Test for Isolation by Distance

**Figure 1:**
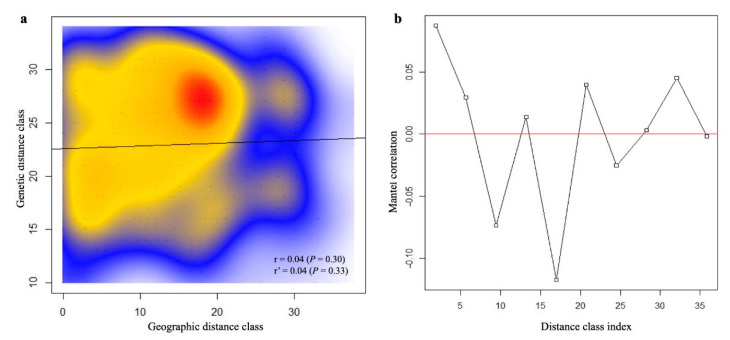
Mantel test of *Pyrus calleryana* dataset. Mantel test (**a**) with Mantel correlogram (**b**) for isolation-by-distance analyses of samples within the *P. calleryana* dataset. The correlation between geographic and genetic distance for the dataset was determined using 1000 permutations at α = 0.05. Distance class indices (in 100 s of km) indicate that the maximum linear distance between samples was 3600 km. Significance (α = 0.05) is reported for the Mantel index (r) and the Mantel index standardized by the year of sampling (r’).

Several independent analyses were performed to determine the population structure in the *P*. *calleryana* collection. Isolation by distance analysis using the Mantel test yielded no evidence of correlation between genetic and geographic distances among the analyzed *P*. *calleryana* individuals of Asian origins (total *n* = 28; Supplementary Figure S1; Mantel statistic (r) = 0.04, *p*-value = 0.30) (Figure 1). The partial Mantel test (standardized geographic distance matrix by the year of sample collection) did not change that result (Mantel statistic using year of sample collection (r’) = 0.04, *p*-value = 0.33). Additionally, there was a non-linear relationship of the genetic and geographic distances across the space (Figure 1b). The amplitude of the Mantel’s r scores in the correlogram was low (−0.1 to 0.05), indicating a low impact of spatial distancing on the population structure of *P*. *calleryana* dataset.

##### Bayesian Clustering Using Structure and DAPC

**Figure 2:**
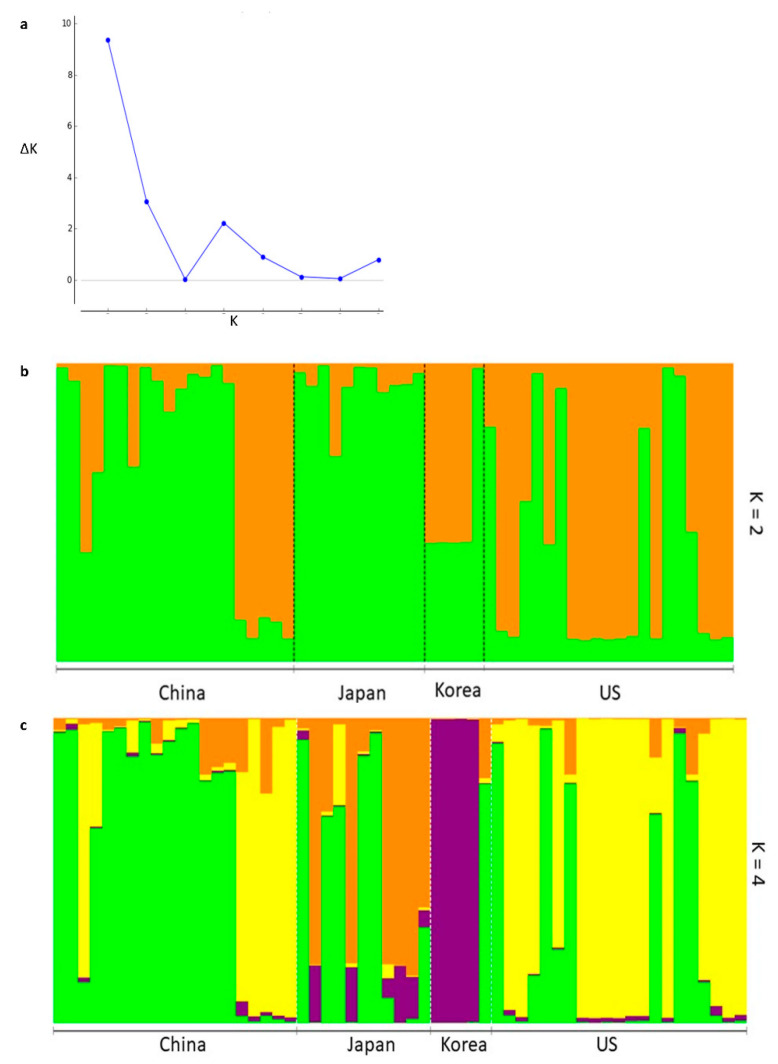
Structure Bayesian clustering of *Pyrus calleryana* dataset. Structure Bayesian clustering analyzed with (**a**) the Evanno method and visualized using (**b**) 2 genetic clusters or (**c**) 4 genetic clusters. Each vertical bar represents an individual sample and the bar color indicates the probability of an individual to get assigned to one of the identified clusters.

Bayesian clustering using Structure indicated two genetically distinct clusters (ΔK = 2) in the genotyped *P*. *calleryana* dataset (Figure 2a,b). The admixture of both the inferred clusters was observed for all four population groups. Additionally, four genetically distinct clusters (ΔK = 4) of the genotyped *P*. *calleryana* dataset (Figure 2c) showed the admixture of all four inferred clusters in all population groups except “Korea”. Negligible variability among 30 independent Markov chains of Structure was detected. The overall R^2^ between predefined and inferred clusters of the *P*. *calleryana* collection (when K = 2) was 0.42 ± 0.07 suggesting moderate divergence among the predefined populations and Structure-derived genetic clusters within the dataset. The information on the contribution of sampled and inferred populations to the observed structure of *P*. *calleryana* was derived by changes to R^2^ using iterative successive removal of the pre-defined populations and the inferred clusters using ObStruct (Supplementary Table S6). Only minor changes in R^2^ index value were evident when the predefined populations or the inferred clusters were removed sequentially. Removal of the “China” population caused decrease (*p-*value = 0.32) in R^2^ indicating that this population contributed the most to the population structure from among those analyzed. Additionally, the removal of the “Japan” population caused increase (*p-*value = 0.20) in R^2^ which suggests that this population was of mixed ancestries and contributed the least to the population structure. Furthermore, removal of the inferred clusters resulted in no major changes in R^2^ indicating no major contribution of the inferred clusters to the structure within the data. In addition to this, the overall R^2^ between predefined and inferred clusters of the *P*. *calleryana* dataset (when K = 4) was 0.41 ± 0.02, again with no major changes when sequentially removing the pre-attributed or the inferred groups (Supplementary Table S6).

**Figure 3:**
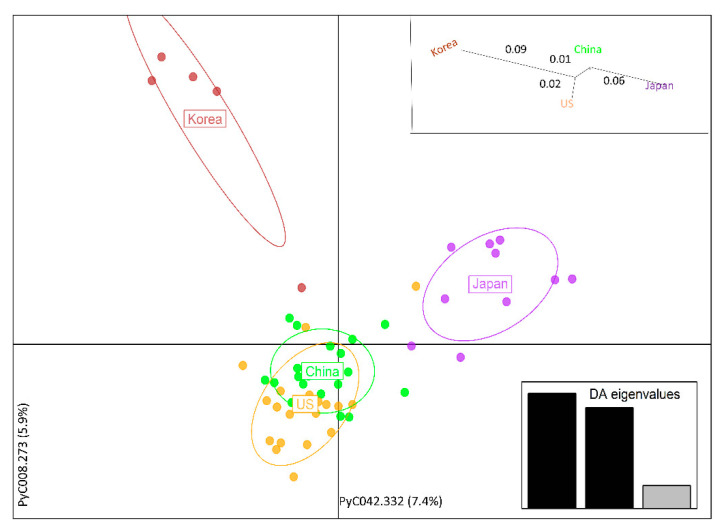
DAPC of *Pyrus calleryana* dataset. DAPC for determining the molecular variance partitioning projected using 15 Principal Components cross-checked and optimized with 1000 permutations. Eigenvalues (*Insert* bar graph, bottom right) represent the factor by which eigenvectors are scaled, which expresses the spatial relationship among populations at different spatial scales. The two respective axes are indicated by the alleles explaining the most of variance within the sampled population. *Insert* genetic distance tree, top right: Unrooted neighbor-joining tree of pairwise genetic distances (F_ST_) [66] among the sampled *P*. *calleryana* specimens.

Using DAPC, a multivariate analysis, the *P*. *calleryana* dataset showed the clustering different from Structure. DAPC indicated 4 clusters for the *P*. *calleryana* dataset. The DAPC result indicated the possibility of the “China” population being ancestral to the bulk of the species with diverged “Japan” and “Korea” populations (Figure 3). This was also supported by the genetic distance dendrogram, and in congruence with the results of the Bayesian clustering. In addition to this, DAPC analysis of USA cultivars alone showed that the cultivars named same from the same or different source institutions were not identical (Supplementary Figure S7).

## 4. Discussion

We investigated the genetic diversity of *P*. *calleryana* in the collection of original non-cultivated Asian specimens and early developed USA cultivars. Available historical records and our results support the hypothesis for high genetic diversity within Asian pear specimens and their genetic relatedness with the early developed USA cultivars. Sample collection from herbaria and arboreta demonstrate their great value for research studies such as ours, or when sampling in native environment is hindered. We found high levels of genetic diversity within *P*. *calleryana* populations supporting the fact that wild populations of forest tree species maintain high levels of genetic diversity [73]. Our findings on *P*. *calleryana* diversity are also supported by other studies of woody forest trees: North America-native *Cercis canadensis* [74] and Asia-native *Cornus kousa* [75].

We developed and used 18 gSSRs discriminating individual MLGs of *P. calleryana*. Despite availability of the SSRs published by others [36], we chose to develop new markers, as is standard in our laboratories [25,75]. This approach increases the pool of available markers and provides a higher level of confirmation for the results accrued by others. Our novel gSSRs also cross-amplified to conserved sequences of DNA extracted from several *Pyrus* species. However, these gSSRs did not perform well, with fewer gSSRs amplifying informative sequences from the more distantly related *Malus* species. Wide genetic variation among *Pyrus* species exists, and the failure of gSSRs to cross-amplify some *Pyrus* species could be due to speciation and changing genomic landscape [36]. Those non-amplified *Pyrus* species are related to *P. bretschneideri*/*P. calleryana* but they are highly admixed likely due to inter-species hybridization and genetic admixture common in *Pyrus* [76]. Nevertheless, the overall cross-amplification potential of our gSSRs makes these novel markers useful for future studies of at least several species of *Pyrus*. Owing to limited resources we did not confirm the gSSR transfer to other species used here; PCR product size agreement provides clues that our cross-amplification was indeed successful. The gSSRs can be sequenced as needed for each species undergoing analyses.

The genetic diversity, allelic richness, and gene flow patterns of any plant species are related to their ecology and evolution [77,78,79]. In an outcrossing and widespread species, the genetic diversity mainly exists within populations [80]. Our study revealed a high level of genetic diversity (H_e_ = 0.81) and allelic richness, and the presence of population structure in *P. calleryana*. A similarly high level of genetic diversity was found in *P*. *calleryana* (H_e_ = 0.639) using 14 nSSRs to study 77 individuals [36] and in *Ambrosia artemisiifolia* (H_e_ = 0.776) using 13 gSSRs and 13 expressed sequence tag SSRs (EST-SSRs) to study 321 individuals [81]. We found high genetic diversity for *P*. *calleryana* populations compared to other invasive species such as southwestern Puerto Rico’s invasive tree [82], *Albizia lebbeck* with H_e_ = 0.27, or southeastern US invasive species [83], *Pueraria lobata* with H_e_ = 0.25.

We recorded high gene flow among *P*. *calleryana* populations across the species’ native range. Irrespective of the geographical barriers, high gene flow could be a possible reason for this species’ invasiveness because gene flow promotes evolution through the spread of new genes or mixture of genes throughout the range of species [84]. The high gene flow rate observed here is consistent with other invasive species [82,85] such as *A. lebbeck* and *Fallopia* species. Such a high gene flow rate promotes exchange of genes among populations especially in self-incompatible species by providing seeds for more population growth and colonization of new habitats [82]. As an outcrossing species, *P. calleryana* has been able to maintain population structure with high genetic diversity. The individuals of a particular cultivar have the same self-incompatibility genotype and cannot produce fruits. However, if the rootstock is allowed to sprout then that rootstock can cross with genetically different scion resulting in fruit set with mixed cultivar types [12]. Additionally, our study found a low impact of spatial distance in population structure of *P*. *calleryana* dataset which could support the seeds or pollen as the far-reaching mechanism of dispersal.

In *P*. *calleryana*, flowers are indiscriminately visited by various generalist pollinator species and the pollen is often carried among neighboring cultivars, resulting in intraspecific hybridization [8]. Seeds can be dispersed over long distances as a result of frequent frugivorous animal activities [34]. In a previous study, most of the *P*. *calleryana* seedlings were found with almost no genetic similarity to nearby mature trees, implying that those seedlings might have originated from foraging birds’ defecation [12]. In addition, there could be an intraspecific hybridization among the cultivars and an interspecific hybridization with the other escaped *Pyrus* species [13], as implied by our cross-amplification data. These characteristics may help *P*. *calleryana* maintain high genetic diversity and high gene flow rate, aiding in the continued spread of the species by providing environment-specific genotypes needed to adapt to a varied environmental condition [82].

High genetic differentiation found in our *P*. *calleryana* collection suggested the existence of population structure. Pears are expected to undergo random mating, but an unexpected positive result for the inbreeding coefficient (F_IS_) values was found in our study indicating the probability of alleles coming from a common ancestor. A similar positive F_IS_ result was found in a study in *P*. *calleryana* in China [36], where the species is considered threatened. One of the possible explanations for such positive F_IS_ result could be that the sampled individuals might have experienced some human interferences, such as selection, propagation, and intentional transportation resulting in the escape of cultivation in the USA. Other explanations could be the insect pollination with limited pollen flow creating local population structure, or the selection of loci under negative selection by chance, where any mutation would be lethal. Studies testing this phenomenon in the escaped *P*. *calleryana* populations are underway.

Our study did not indicate geographic distance as the major factor contributing to the genetic structure of the populations. A non-significant correlation between genetic and geographic distance could be a result of the small size of our *P*. *calleryana* dataset collection. Our result is unlike the Mantel test result obtained for wild *P*. *calleryana* from China [36]. In addition to this, our study partitioned 57 individuals from 4 populations into 2 major genetic clusters. However, considering the biology of *P*. *calleryana*, DAPC result, unrooted neighbor-joining tree, and bias of Structure towards k = 2 [86,87], we assumed k = 4 as the best clustering for our *P*. *calleryana* dataset. Almost all *P*. *calleryana* individuals throughout all populations showed extensive genetic admixture, in agreement with the detected high gene flow among populations. The individuals from “China” and “USA” population groups had relatively higher assignment probability to the major genetic clusters. Furthermore, genetic distances of “China” and “USA” populations placed them close to each other. Thus, our results support the historical import records of *P*. *calleryana* from China to the USA [12] with the “China” population being ancestral to the “USA” population.

The historical events suggest the development of *P*. *calleryana* cultivars using *P*. *calleryana* as a common rootstock. We expected at least some clones within the “USA” *P*. *calleryana* individuals as such cultivars were multiplicated clonally. However, no clones within the “USA” *P*. *calleryana* individuals were found, as each “USA” *P*. *calleryana* individual was unique with no shared MLGs within this population. Our study also found no identical cultivars although named the same from the source institutions. In such case, ‘Trinity’ cultivar of one source is genetically different from ‘Trinity’ cultivar of another source. Such a great genetic composition and diversity with no clones in our “USA” *P*. *calleryana* individuals signifies the great invasive potential of the species, and alarms about the level of the previously noted cultivar mislabeling and mishandling [7,88]. Our result is in stark contrast to those of others, who used 2 SSRs from *P*. *pyrifolia* and 7 from *Malus* × *domestica* on 14 *P*. *calleryana* cultivars [7]. They detected clonal MLGs in agreement with the cultivar description, confirming genetic identity of ‘Chanticleer’, ‘Cleveland Select’, and ‘Stonehill’- in accord with their history, and origin from the same source [7].

The high level of genetic diversity within the investigated *P*. *calleryana* collection suggests the high potential of the species for evolving resilience and ability to thrive in a variety of environmental conditions. In addition, the high gene flow among *P*. *calleryana* populations creates the complex genetic admixture, adding more challenge to their control. For the development of measures for improved control, it is necessary to study in detail the genetic composition and diversity, and to infer the evolutionary potential of the species across the introduced ranges. Considering the ultimate goal of well-informed control measures, we are already taking the next steps in analyses of the naturalized *P. calleryana*: the fine-scale study of *P. calleryana* in localized area of the USA using the gSSRs developed here, and the broad-scale study of *P. calleryana* across the USA using reduced-representation genotyping-by-sequencing. Thus, *P*. *calleryana* populations across the USA provide a great prospect for further research and study, to better understand the spread mechanism of this invasive species.

## 5. Conclusions and Outlook

Our study has highlighted factors in *P*. *calleryana* native populations that are likely to be contributing to its invasive capability within introduced range in the USA. This tree species has been able to maintain high genetic diversity, which has helped it survive and adapt under diverse environmental, physical, and geographical conditions. The genetic complexity of the species adds complications to efforts at managing naturalized populations and mitigating further spread. The evidenced mishandling of the cultivars likely contributed to enabling the species spread in the naturalized range. In order to limit the cultivar mishandling, we suggest nursery professionals to verify their cultivars with the original ones or to maintain only one/few properly identified cultivars per station, and to consider the high genetic diversity of *P*. *calleryana* in large scale production of *P*. *calleryana* saplings. Further molecular and remote imagery research is underway to better understand the mechanisms behind the observed spread of *P*. *calleryana* across the USA.

## Figures and Tables

**Table 1:** Genetic diversity indices of *P*. *calleryana* dataset on the basis of population groups using 18 gSSR loci.

Population	N	% Missing	# Alleles	NAe	H	G	F_IS_	A_r_	λ	H_e_	H_o_	$\bar{r}$_d_	P_a_
China	20	15.28	8.06	6.40	3.00	20	0.636 ^***^	4.61	0.95	0.80	0.30	0.10 *^ns^*	37
Japan	11	5.56	5.72	4.74	2.40	11	0.511 ^***^	4.02	0.91	0.74	0.37	0.16 ^***^	19
Korea	5	7.78	2.56	2.41	1.61	5	0.569 ^***^	2.48	0.80	0.51	0.24	0.03 *^ns^*	10
USA	21	7.94	7.22	4.62	3.04	21	0.397 ^***^	4.06	0.95	0.72	0.44	0.11 *^ns^*	22
Total	57	10.04	11.78	4.54	4.04	57	0.552 ^***^	3.79	0.98	0.80	0.36	0.12 *^ns^*	88

N: Number of individual samples used; % Missing: percent of genotypes missing; # Alleles: Number of alleles present in the given population group (calculated using SPAGeDi); NAe: Effective number of alleles present in the given population group [68]; H: Shannon-Wiener Index of MLG diversity [69]; G: Stoddart and Taylor’s Index of MLG diversity [70]; F_IS_: Individual inbreeding coefficient (^***^ = *p* < 0.0001); A_r_: Allelic richness giving the expected number of alleles among 8 gene copies; λ: Simpson’s Index [71]; H_e_: Nei’s unbiased gene diversity [66]; H_o_: Observed heterozygosity; $\bar{r}$_d_: Standardized index of association taking into account for the number of loci sampled [24]; P_a_: Number of private alleles in each population group. Significance was assessed by using 10,000 randomizations of gene copies among all individuals, ^***^ = *p* < 0.0001 and *^ns^* = *p* > 0.05 at 10,000 permutations.

**Table 2:** Population genetics indices of *P*. *calleryana* dataset on the basis of microsatellite loci.

SSR Locus	No.	% Missing	A_r_	H_o_	H_e_	D_est_	G	F_ST_	F_IS_	R_ST_	R_IS_	N_m_
PyC006	10	12.28	3.89	0.13	0.72	0.20	3.53	0.08 *^ns^*	0.74 ^****^	0.18 ^*^	−0.08 *^ns^*	5.58
PyC008	16	3.51	4.86	0.36	0.87	0.82	7.36	0.16 ^****^	0.38 ^****^	0.47 ^****^	0.69 ^***^	0.85
PyC009	15	12.28	5.39	0.25	0.85	−0.14	6.13	−0.02 *^ns^*	0.72 ^****^	0.18 ^*^	0.70 ^****^	NA
PyC012	13	26.32	4.73	0.08	0.89	0.78	8.34	0.14 ^***^	0.85 ^****^	0.17 *^ns^*	0.85 ^****^	1.07
PyC013	14	0.00	4.34	0.43	0.84	0.50	5.86	0.11 ^***^	0.46 ^****^	0.11 ^*^	0.36 ^*^	1.23
PyC014	14	7.02	5.45	0.21	0.86	0.72	6.84	0.16 ^****^	0.70 ^****^	0.50 ^****^	0.67 ^****^	0.94
PyC015	18	5.26	5.40	0.12	0.87	0.85	7.03	0.20 ^****^	0.85 ^****^	0.74 ^****^	0.87 ^****^	0.67
PyC017	20	21.05	6.48	0.60	0.92	0.46	10.80	0.06 ^**^	0.19 ^**^	0.18 ^*^	0.37 ^*^	3.34
PyC018	16	8.77	5.89	0.65	0.91	0.51	10.18	0.05 ^**^	0.17 ^**^	−0.04 *^ns^*	0.09 *^ns^*	3.20
PyC020	13	0.00	4.89	0.39	0.80	0.20	4.82	0.03 *^ns^*	0.49 ^****^	0.07 *^ns^*	0.34 ^*^	5.50
PyC031	20	8.77	6.22	0.84	0.92	0.57	11.76	0.04 ^*^	0.13 ^*^	0.03 *^ns^*	−0.38 ^**^	6.86
PyC032	6	19.30	4.24	0.09	0.74	0.58	3.77	0.22 ^****^	0.86 ^****^	0.37 ^***^	0.73 ^****^	0.93
PyC035	13	1.75	4.77	0.41	0.85	0.31	6.54	0.03 *^ns^*	0.49 ^****^	0.14 ^*^	0.17 *^ns^*	7.91
PyC038	6	0.00	4.06	0.93	0.76	−0.04	4.12	−0.01 *^ns^*	0.23 ^***^	0 *^ns^*	−0.75 ^****^	NA
PyC041	8	14.04	4.08	0.29	0.81	0.04	5.04	0.01 *^ns^*	0.65 ^****^	0.08 *^ns^*	0.22 ^****^	7.04
PyC042	7	15.79	3.57	0.06	0.81	0.70	5.08	0.23 ^****^	0.89 ^****^	0.62 ^****^	0.82 ^****^	0.61
PyC047	6	12.28	3.64	0.18	0.76	0.36	3.97	0.10 ^*^	0.61 ^****^	0.19 ^*^	0.75 ^****^	2.36
PyC050	5	12.28	2.74	0.03	0.41	0.07	1.68	0.10 *^ns^*	0.90 ^****^	0.09 *^ns^*	0.92 ^****^	2.74
Average	12.2	10.04	4.70	0.34	0.81	0.42	6.27	0.09 ^****^	0.52 ^****^	0.33 ^****^	0.31 ^****^	1.79

No.: Number of alleles detected; % Missing: % of samples that failed to amplify in the given locus; A_r_: Allelic richness; H_o_: Observed heterozygosity (Frequency of heterozygous individuals per locus averaged over the number of sampled loci); H_e_: Expected heterozygosity (Nei’s unbiased gene diversity [66]); D_est_: Jost’s differentiation estimate [72]; G: Stoddard and Taylor index [70]; F_ST_: a measure of sub-population genetic structure; F_IS_: a measure of deviations from Hardy–Weinberg equilibrium in terms of heterozygote deficiency if <0 or homozygote excess if >0; R_ST_ and R_IS_: Analogues of F_ST_ and F_IS_ based on allele sizes [54]; Nm: Gene flow estimated as Nm = ¼ × [(1/FST) − 1]. Significance of the dataset was assessed by 10,000 randomization of gene copies among all individuals using ^****^ = *p* < 0.0001; ^***^ = *p* < 0.001; ^**^ = *p* < 0.01; ^*^ = *p* < 0.05; *^ns^* = *p* > 0.05. NA: Not applicable as a negative value does not represent any gene flow data.

## Data Availability

All data generated during this study are available in the Supplementary Information.

## Supplementary Materials

The following are available online at https://www.mdpi.com/article/10.3390/life11060531/s1, Supplementary Supplementary Figure S1. Map of geographic origins for the Asian samples of *P. calleryana* with known details (*n* = 28). Locations for several specimens are obscured due to the marker size and the overlap. Map generated using GoogleEarth version 7.3.3.7786 (Google LLC, Mountain View, CA, USA), with surface distance of 300 km marked. Supplementary Figure S2. Frequency of di-, tri-, tetra-, and hexa-nucleotide motif repeats disovered in 306 single motifs polymorphic gSSRs collection. The indicated motifs and their redundant iterations are grouped together. *Insert*: Frequency of di-, tri-, and tetra-nucleotide motif repeat gSSRs in the discovered 105,557 gSSRs collection; bp = base pair. Supplementary Figure S3. Gel image of cross-species amplification using ITS and *rps*16, ran on the same gel. (**a**) The uppermost row in gel image represents the amplification done using ITS primers [45]; (**b**) The lowermost row in gel image represents the amplification done using *rps*16 primers [46]. Order of samples in (**a**) and (**b**) is identical, and fro\m left to right includes: DNA ladder (DNA molecular marker of 100 bp; BIONEER, Catalog No.:D-1030; Oakland, California, USA); positive control (*P. calleryana*; PC_A_019); negative control (water); *P. gharbiana* (20_PC_AO_29 and 20_PC_AO_33); *P. korshinskyi* (20_PC_AO_14 and 20_PC_AO_15); *P. regelii* (20_PC_AO_16 and 20_PC_AO_21); *P. hybrid* (‘Bartlett’ × *P. salicifolia*; 20_PC_AO_08). The expected size of PCR products for ITS was 678 bp and for *rps*16 was 911 bp. Supplementary Figure S4. Hardy–Weinberg Equilibrium observed for samples included within the *P. calleryana* dataset. The rows represent loci and columns represent populations. The legend represents the probability of loci to follow HWE. The loci in pink are suspected of not being in HWE with *P* ≤ 0.05. Supplementary Figure S5. Genotype Accumulation Curve (GAC) for samples included within the *P. calleryana* dataset. It represents the number of MLG detected (Y-axis) in relation to the number of loci (X-axis) used for genotyping. Supplementary Figure S6. Pairwise linkage disequilibrium (LD) among the 18 gSSRs for samples included within the *P. calleryana* dataset. It is expressed as standardized index of association ($\bar{r}$_d_). Legend values coincide with hues that explain the strength of the linkage calculated between each pair of the markers. Supplementary Figure S7. DAPC of *P*. *calleryana* USA cultivars from different source institutions. DAPC for determining the molecular variance partitioning projected using 15 Principal Components cross-checked and optimized with 1000 permutations. The two respective axes are indicated by the alleles explaining the most of variance within the sampled population. Each different color represents the different source institutions. Each colored dot represents *P*. *calleryana* USA cultivar individuals. The names in the figure indicate the following *P*. *calleryana* cultivars: Ari01: ‘Aristocrat’; Br01-Br08: ‘Bradford’; Ca01: ‘Cambridge’; Ch01 and Ch02: ‘Chanticleer’; Hm01 and Hm02: ‘Holmford’; Re01: ‘Redspire’; Tr01 and Tr02: ‘Trinity’; and US034 and US035: Unknown cultivars. Table S1. Callery pear samples used for the study, Supplementary Table S2. gSSRs used for the study of *P*. *calleryana,* Supplementary Table S3. Genotyping dataset used for the study (binned and converted to repeat number, Supplementary Table S4. Cross-species amplification of the studied gSSRs, Supplementary Table S5. Analysis of Molecular Variance of *P*. *calleryana* dataset, Supplementary Table S6. R^2^ value using Obstruct).
